# Hypothetical protein predicted to be tumor suppressor: a protein functional analysis

**DOI:** 10.5808/gi.21073

**Published:** 2022-03-31

**Authors:** Md. Abdul Kader, Akash Ahammed, Md. Sharif Khan, Sheikh Abdullah Al Ashik, Md. Shariful Islam, Mohammad Uzzal Hossain

**Affiliations:** 1 Department of Biotechnology and Genetic Engineering, Mawlana Bhashani Science and Technology University, Tangail 1902, Bangladesh; 2 University of Kentucky, Lexington, KY 40506, USA; 3 Bioinformatics Division, National Institute of Biotechnology, Dhaka 1349, Bangladesh

**Keywords:** functional annotation, hypothetical protein, novel bacterium, tumor suppressor, VHL domain

## Abstract

*Litorilituus sediminis* is a Gram-negative, aerobic, novel bacterium under the family of Colwelliaceae, has a stunning hypothetical protein containing domain called von Hippel-Lindau that has significant tumor suppressor activity. Therefore, this study was designed to elucidate the structure and function of the biologically important hypothetical protein EMK97_00595 (QBG34344.1) using several bioinformatics tools. The functional annotation exposed that the hypothetical protein is an extracellular secretory soluble signal peptide and contains the von Hippel-Lindau (VHL; VHL beta) domain that has a significant role in tumor suppression. This domain is conserved throughout evolution, as its homologs are available in various types of the organism like mammals, insects, and nematode. The gene product of VHL has a critical regulatory activity in the ubiquitous oxygen-sensing pathway. This domain has a significant role in inhibiting cell proliferation, angiogenesis progression, kidney cancer, breast cancer, and colon cancer. At last, the current study depicts that the annotated hypothetical protein is linked with tumor suppressor activity which might be of great interest to future research in the higher organism.

## Introduction

Bacteria possess tremendous compatibility that can be used to the necessity of human welfare and *Litorilituus sediminis* can be one of them. *L. sediminis* is a Gram-negative, aerobic, curved-rod shaped, non-spore-forming, catalase, and oxidase-positive bacterium with the polar or sub-polar flagellum. It was isolated from a sediment sample that was collected from the coastal region of Qingdao, China [1]. This organism grew optimally at 37°C, pH 8–9. This type of bacterium was novel among the other genera under the family of Colwelliaceae. The characteristics like phenotypic, chemotaxonomic, and well-confirmed phylogenetic evidence of *Litorilitus* belonging to the family Colwelliaceae was distinctive that implied as a novel genus. This novel bacterium has a prominent concentration of cellular constituents compared with other genera and these are C16:0 and C16:1 ω7c fatty acids, phosphatidylethanolamine, phosphatidylglycerol, aminophospholipid, and two amino lipids (AL1, AL2) as well as isoprenoid quinone 8 [1]. Along with bacterial cellular components, a profuse number of proteins exist where approximately 2% of the genes code for proteins as well as the remaining are non-coding or still functionally unknown [2].

The number of genes having unknown functions referred to as hypothetical proteins is present in each organism’s genome [3] and these are a category of the protein whose existence is not confirmed by any experimental evidence but can be predicted to be expressed from an open reading frame [4]. The hypothetical proteins can be classified as uncharacterized protein families which are experimentally verified to exist but have not been identified or linked to a known gene, and the other type is the domain of unknown functions [5] that is experimentally characterized proteins in the absences of known functional or structural domains [6,7]. Despite the lack of functional characterization, they play a significant role in understanding biochemical and physiological pathways like exploring new structures and functions [8], pharmacological targets and markers [9], and early detection and benefits for proteomic and genomic research [10]. With the advancement of Computational Biology, it has become easier to analyze hypothetical proteins using bioinformatics tools that provide various advantages like the determination of 3D structural conformation, identification of new domains and motifs, assessment of new cascades and pathways, phylogenetic profiling, and functional annotation [11]. A recent study showed that the annotated hypothetical protein is linked with hydrolase activity which might be of great interest to further research in bacterial genetics [12].

However, due to novel genera under the family of Colwelliaceae, this study intended to characterize the protein EMK97_00595 (*Litorilituus sediminis*), a family of von Hippel-Lindau (VHL) that have an overwhelming function as a tumor suppressor in higher organisms. The main feature of VHL is that it is a critical regulator of the ubiquitous oxygen-sensing pathway and can act as a substrate recognition component of an E3 ubiquitin ligase complex [13], also promote the degradation of epidermal growth factor receptor, pro-angiogenesis factors, remodeling of the extracellular matrix, and helps in apoptosis resulting tumor suppression [14].

In the higher organism during cellular normoxia when oxygen is available, the cellular hypoxia-inducible factor 1α (HIFα) is hydroxylated by prolyl hydroxylase and works as a felicitous substrate for von Hippel-Lindau tumor suppressor protein (pVHL) which is a constitutive active site of E3 ubiquitin ligase. The hydroxyproline of hydroxylated HIFα provides a binding signal for pVHL, which leads to efficient ubiquitylation and proteasomal degradation of HIFα protein. On the other hand, in hypoxia condition HIFα is not prolyl hydroxylated and may escape pVHL recognition, resulting in accumulation of HIFα and formation of a complex with HIF1β, goes into the nucleus and activates a transcriptional program to cope with the short-term, long-term effects of oxygen deprivation, several signaling pathways as well as angiogenesis factor for leading cell proliferation or tumor [14,15]. So the function of the hypothetical protein that exists in the *L. sediminis* is considerable.

Therefore, this study manifests a reliable interpretation of this hypothetical protein EMK97_00595 (QBG34344.1) by adopting an integrated workflow that can be a potential research interest in the field of tumor suppression study.

## Methods

### Sequence retrieval and similarity identification

The hypothetical protein EMK97_00595 (*Litorilituus sediminis*) was chosen by exploring the NCBI database which can act as a significant research interest in numerous cancer research fields in the near future (Supplementary Table 1). The sequence of the hypothetical protein (GenBank accession: QBG34344.1 and NCBI reference sequence: WP_130598461.1) that may contain a tumor suppressor domain was retrieved and collected as a FASTA format and submitted to several prediction servers for the in-silico characterization. Initially, a similarity search was performed using the NCBI BLASTp program [16] against the non-redundant and Swissprot database [17], for predicting the function of the hypothetical protein.

### Multiple sequence alignment and phylogeny analysis

A multiple sequence alignment is a tool used to explore closely related genes or proteins to find the evolutionary relationships between genes and to identify shared patterns among functionally or structurally related genes. Sequence alignment was performed by the MUSCLE server of EBI [18], and an evolutionary relationship was accomplished by Jalview 2.11 software [19], between the hypothetical protein EMK97_00595 and the proteins that had structural similarity with the protein of interest.

### Analysis of physicochemical properties

ProtParam [5] is a tool that computes various physical and chemical parameters of protein sequences. The physicochemical properties of the hypothetical protein were predicted using the ProtParam tool in the ExPASy server [20], which predicts all the relative properties including molecular weight, theoretical pI, amino acid composition, the total number of positive and negative residues, instability index, aliphatic index and grand average of hydropathicity (GRAVY) [21-23].

### Analysis of the secondary structure

The servers that were utilized to predict protein secondary structure were SOPMA [24] and PSIPRED [25]. SOPMA is a general secondary structure prediction tool, on the other hand, PSIPRED is a server for comprehensive analysis of protein. The server SOPMA was initially employed to predict the secondary structure and then the result derived from the SOPMA server was validated by exploiting PSIPRED.

### 3D structure modeling and quality assessment

HHpred server [26] that works based on the pairwise comparison profile of hidden Markov models, was used to build the 3-dimensional structure using the best scoring template. The confidence of the predicted structure was also visualized by SWISS-MODEL [27]. Several quality assessment tools of the SAVES and ProFunc [28] server were applied to estimate the reliability of the predicted 3D structure model of the hypothetical protein. The Ramachandran plot for the model was built using the PROCHECK program [29] to visualize the backbone dihedral angles of amino acid residues. The quality of the protein 3D structure was assessed with the help of the ERRAT server [30] and Varify 3D server was used to determine the compatibility of an atomic model (3D) with its amino acid sequence as well as comparing the results to standard structures [31,32].

### Active site determination

Computed Atlas of Surface Topography (CASTp) is an online active site determination server [33] that calculates the location, delineation, and concave surface regions on 3D structures of proteins. CASTp predicted the active site of the selected hypothetical protein that showed the binding sites, amino acid binding regions with area and volume.

### Identification of protein subcellular localization and topology

The subcellular location of the following protein was predicted by using the BUSCA web server [34]. BUSCA amalgamates different tools—DeepSig, TPpred3, PredGPI, BetAware, ENSEMBLE3.0, BaCelLo, MemLoci, and SChloro to predict protein features related to localization. The result was further checked by Cello [35], PsortB [36], Gneg-mPLoc [37], SOSUIGramN [38], and PSLpred [39]. Prediction of signal peptide was done by using PrediSi [40] and SignalP-5.0 Server [41]. The solubility of the hypothetical protein was evaluated by Protein-sol [42] and SOSUI [43] webserver. Protein transmembrane helices were assessed by HMMTOP [44], TMHMM [45], and Sable [46] webserver. The topology of hypothetical protein was predicted by the ProFunc server [14].

### Prediction of protein domain, superfamily, family, coil, and folding pattern

Domain/superfamily/family of the following hypothetical protein was analyzed by using the servers—CDD (conserved domain database) from NCBI [47], Pfam [48], SMART [49], Interpro [50], SCOP [51,52], Supfam [53], Motif, ProFunc [28], Phyre [54], and CATH-Gene3D [55]. Among them, CDD, Pfam, SMART, Interpro, SCOP, Supfam, MotifFinder were employed to predict function from the sequence of the hypothetical protein, and ProFunc, Phyre 2, and CATH-Gene3D servers were used to predict the function from the 3-dimensional structure of the hypothetical protein. Only the lowest e-value was considered to determine protein classification, which indicates good similarity. The protein folding pattern was determined by using Phyre 2 and PFP-FunDSeqE [56] servers where protein coil nature was determined by using PCoils [57] from the Bioinformatics toolkit server.

### Generation of protein-protein interaction network

As the proposed investigation seeking a tumor suppressor protein from microorganisms, STRING [58] has been used to summarize the network information of VHL tumor suppressor protein. Because of being a novel microorganism, there is no specific network is available. Here the VHL protein from humans has been used as a supposition model that might give an intellectual knowledge about VHL protein if it may apply to the human.

## Results

### Identification of sequence homology

**Fig. 1. f1-gi-21073:**
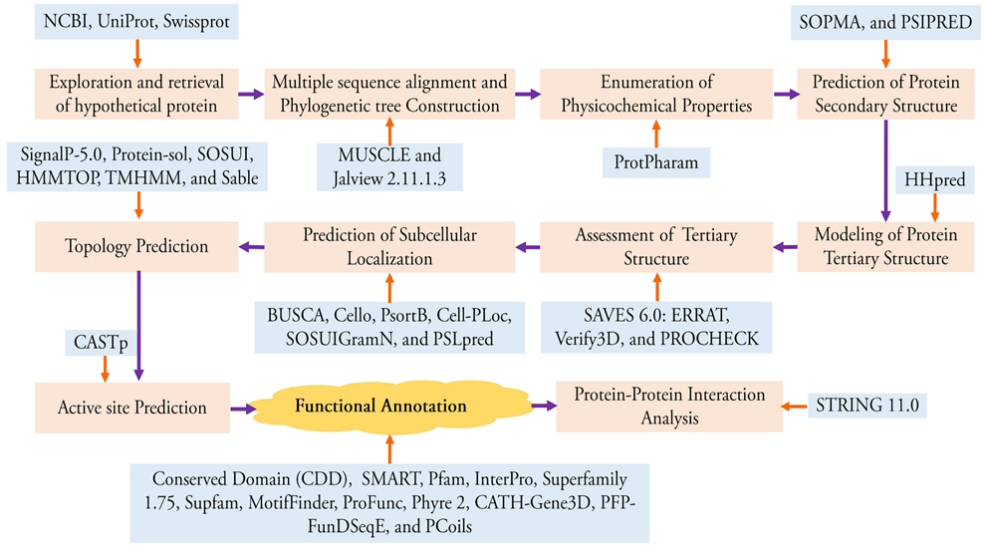
A schematic representation of the overall experimental design.

**Fig. 2.**
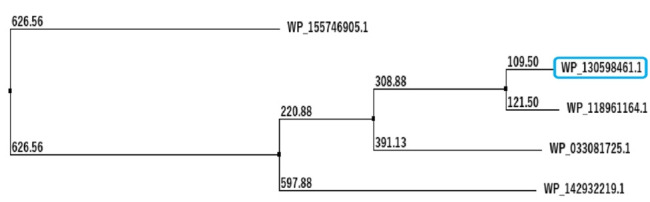
Evolutionary analysis of different von Hippel-Lindau (VHL) proteins with the target protein shown in the blue box (WP_130598461.1). Evolutionary analysis of different VHL proteins with the target protein shown in the blue box (WP_130598461.1) having maximum query cover, score and identity with its close relative *Colwellia* sp. RSH04 (WP_118961164.1) and other organisms. The BLASTp result against non-redundant and SwissProt database showed homology with other von Hippel-Landau (pVHL) domain-containing proteins. Multiple sequence alignment was considered the FASTA sequences of the hypothetical protein (QBG34344.1) and the homologous annotated proteins. Phylogenetic analysis was performed to confirm homology assessment between the proteins, down to the complex and subunit level. The tree was constructed based on the alignment where distances between branches were also included and the BLASTp result gives a similar concept about the protein.

The overall workflow of this study has been shown in Fig. 1. The BLASTp result of the FASTA sequence of the selected protein shows the sequence homology with other identical proteins (Tables 1 and 2). Construction of phylogenetic tree using multiple sequence alignment generated from BLASTp result shows the evolutionary relationship of the selected hypothetical protein (WP_130598461.1) (Fig. 2).

### Analysis of physicochemical properties

The physicochemical properties of a protein can be characterized by an analysis of the analogous properties of the amino acids (Supplementary Table 2). The hypothetical protein is negatively charged as the theoretical pI: 4.22 and the total number of positively (Arg + Lys) and negatively charged residues (Asp + Glu) were found to be 10 and 27, respectively. The computed instability index was 32.71 classifying the protein as a stable one. The aliphatic index was 77.37 which gives an indication of proteins’ stability over a wide temperature range and all the other properties have been summarized (Supplementary Table 2).

### Secondary structure analysis

**Fig. 3.**
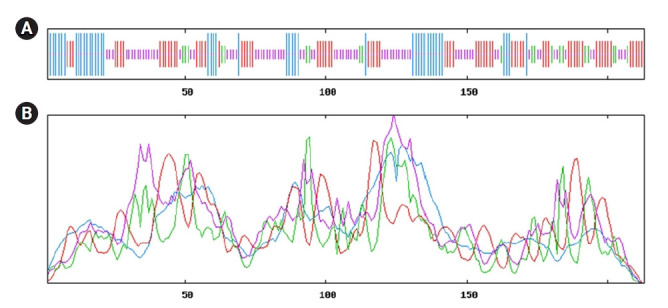
Model of secondary structure. (A) Secondary structure information from SOPMA server. (B) Sequential organization and graphical visualization of secondary structure from PSIPRED.

The secondary structure of a protein can be able to provide some worthy information about the function. The query hypothetical protein shows the percentages of alpha-helix, beta-turn, extended strand, and the random coil of protein 21.13%, 9.91%, 33.33%, and 36.15%, respectively from SOPMA (Supplementary Figs. 1 and 2, Supplementary Table 3). The results of the secondary structure were also cross-checked by the PRISPRED server which shows a summary of similar results (Supplementary Fig. 3). The representative secondary structure of the hypothetical protein (WP_130598461.1) has been shown (Fig. 3).

Secondary structure predicted from SOPMA server directed (Fig. 3A); having maximum portion of random coil (36.15%), extended strand (33.33%) and alpha-helix (21.13%) and others information displayed in Supplementary Fig. 1 and Table 3. Here, alpha-helix, beta-turn, extended strand and the random coil is indicated as blue, green, red and orange, respectively (Fig. 3A). Simultaneous analyses of secondary structure from the PSIPRED server was presented (Fig. 3B, Supplementary Fig. 3), where the helix, strand and coil sections were indicated by specified color code. Other information is available in Supplementary Figs. 2‒6.

### Assessment and validation of protein 3-dimensional structure

**Fig. 4.**
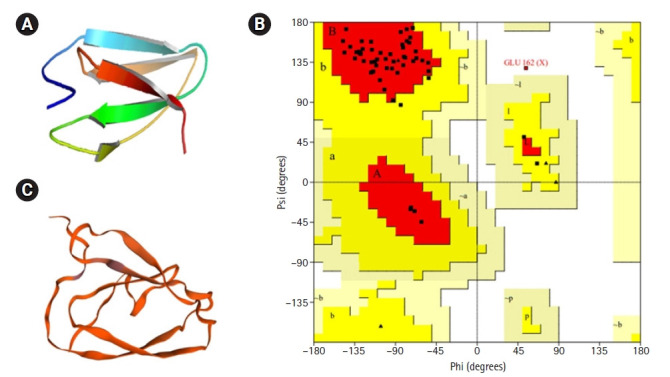
Graphical representation and assessment of protein 3D structure. Predicted 3-dimensional structure from SAVES server (Pymol view) (A), from SWISS-MODEL (B), and Ramachandran plot analysis of 3D modeled structure validated by PROCHECK program (C).

**Fig. 5.**
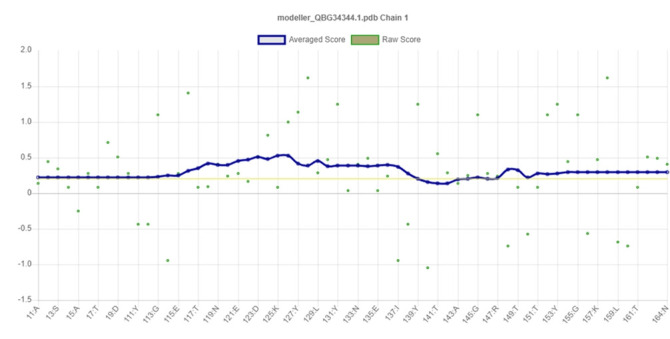
3D-structure validation by Verifiy3D.

PROCHECK program was used for the validation of predicted tertiary structure, where the distribution of φ and ψ angle in the model within the limits are shown (Table 4, Fig. 4). The model was presumed to be a good one according to the Ramachandran Plot Statistics, with 91.1% residues in the most favored regions. Finally, the structure validation server Verifiy3D and ERRAT was implicated in verifying the established model of 3D structure for the target sequence. In the Verify3D graph, 93.75% of the residues have averaged a 3D-1D score ≥ of 0.2 which indicates that the environmental profile of the model is good (Fig. 5)and the overall quality factor predicted by the ERRAT server was 60.7143 indicates a quality model (Supplementary Fig. 7). From ProFunc, the average G-factors of the hypothetical protein is calculated to be ‒0.20, which indicates a usual protein model. Overall quality factor of the structure has been also depicted (Supplementary Fig. 5).

### Active site calculation

**Fig. 6.**
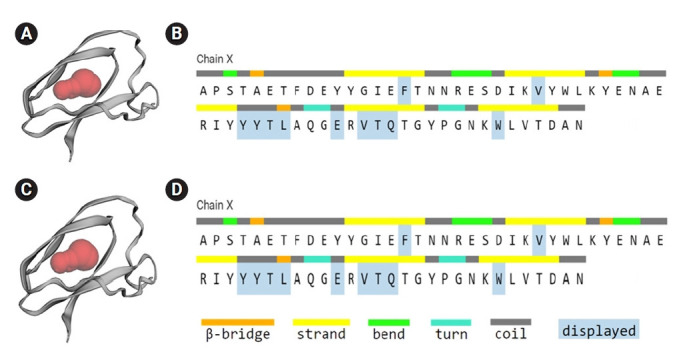
Active site of the hypothetical protein, binding site of the hypothetical protein indicated by red region (A, C), and amino acids involved in the active site (B, D).

The active site of the selected hypothetical protein constituted by 11 amino acids of an area with 52.957 and a volume of 22.609. Chain X of the hypothetical protein shows the amino acids involved in the active site (F, V, Y, Y, T, L, E, V, T, Q, W) (Fig. 6A and 6B).

The selected hypothetical protein has 11 active sites with variable size and is constituted by 64 amino acids demonstrated (Fig. 6A and 6B). Different binding pockets of the hypothetical protein were indicated as red, blue, green, purple, orange, and pink region, and where the amino acids contributing to the beta-bridge, beta-strand, bend, turn, and coiled regions were specified by colored bars. The largest active site (red spheres) with the contributing amino acids was directed (Fig. 6C and 6D).

### Assessment of protein subcellular localization and topology

The subcellular localization of the hypothetical protein seems to be an extracellular secretory signal peptide. Protein-sol and SOSUI both predict the hypothetical protein as a soluble protein. HMMTOP, TMHMM predicted the protein as a non-transmembrane protein (Table 5). The predicted topology of the protein has shown here from N-terminal to the C-terminal.

**Fig. 7.**
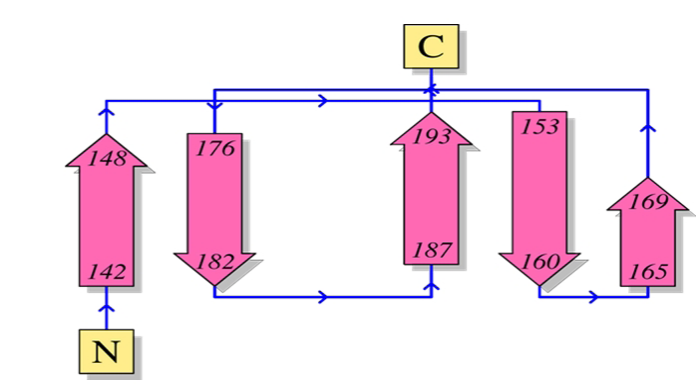
Topology of hypothetical protein.

Topology of the hypothetical protein EMK97_00595. The topological orientation of the respective strands depicted (pink arrow) from the amino terminal (N) to the carboxyl terminal (C) end exposed in Fig. 7.

### Functional annotation of the hypothetical protein

The initial protein domain was achieved from the CDD of NCBI. The region of the domain, superfamily, and family classifications have been determined by the servers—CDD, Pfam, SMART, Interpro, SCOP, Supfam, MotifFinder, ProFunc, Phyre 2, and CATH-Gene3D. The domain, superfamily, and family were selected based on the lowest e-value of the following domain. The higher e-value has been filtered out from the selection procedure. The e-value 9.11e-05 of VHL beta domain from ProFunc, 2.71e-09 of VHL superfamily from SCOP, 8.1e-03 of VHL family from Supfam indicate extremely good protein alignment, respectively. The overall alignment range of the VHL beta domain was 133-212, VHL superfamily and family were 144‒200, respectively. Protein coil nature was determined by using PCoils from the Bioinformatics toolkit server. According to Phyre 2, the folding pattern of the following hypothetical protein is pre-albumin-like. On the other hand, PEF-FunSeqE is called the protein immunoglobulin-like. Both are secreted protein as well as soluble protein and hence provide a properly defined similarity indication of VHL protein (Table 6, Supplementary Fig. 4 and 7-9).

### Analysis of protein network

**Fig. 8.**
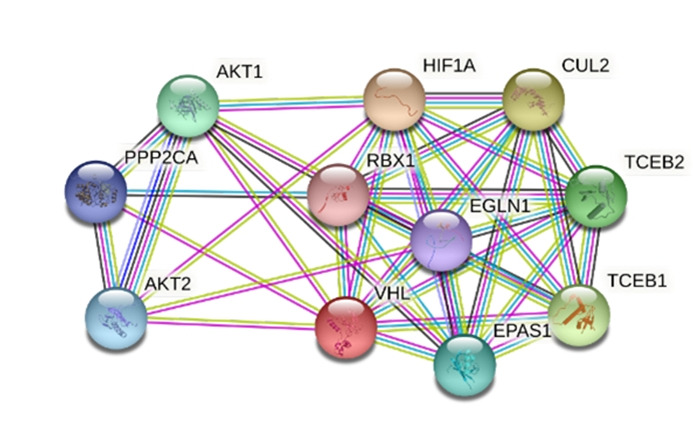
Protein-protein interaction network of the hypothetical VHL protein. VHL, von Hippel-Lindau.

The STRING interaction of VHL protein from *Homo sapiens* has been shown in Fig. 8 as a model. VHL interacts with various proteins based on their combined score (Table 7). The network has 11 nodes, 40 edges, average node degree 7.27, local clustering coefficient 0.819, expected number of edges 18, and the p-value of protein-protein interaction enrichment 7.07e-06 indicates the network has significantly more interactions than expected.

Because of being a noble microorganism that produces hypothetical VHL protein, the VHL protein from humans has been used as a supposition model that likely to be similar to VHL protein found from microorganisms. The model VHL protein interacts with 10 other proteins such as AKT1, AKT2, CUL2, EGLN1, EPAS1, HIF1A, PPP2CA, RBX1, TCEB, and TCEB2.

#### Similarity analysis between query (*Litorilituus sediminis*, EMK97_00595) and target (*Homo sapiens*, AAB64200.1) pVHL proteins

The mentioned *L. sediminis* (EMK97_00595) and target (*Homo sapiens*, AAB64200.1) pVHL proteins (Table 8) molecular weight, aliphatic index, and pI value bolster the confidence value between these two pVHL proteins to be more congruous for their almost resemble value [59].

The other properties like helix, coil, and beta sheet contents are also comparable whereas the beta sheet contents were massive in the query protein rather than target protein which implies that the bacterial query pVHL proteins have higher potentiality to drive role as a tumor suppressor protein comparing with human pVHL proteins. Because the beta domain in the pVHL protein provide the binding site for HIFα degradation. The most intriguing matter from the comparisons, the query protein is highly stable rather than the human protein which implicate to substitute this protein in human is considerable [60].

**Fig. 9.**
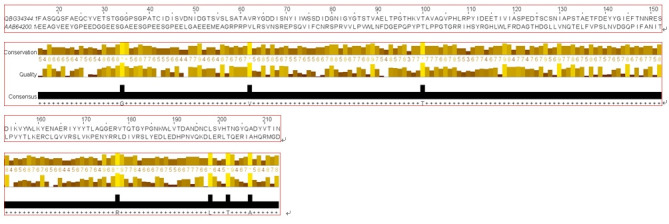
The amino acid sequence alignment between query and target pVHL protein. The black legends below the two amino acid sequences alignment indicate the consensus amino acid of the protein (from Jalview analysis). pVHL, von Hippel-Lindau tumor suppressor protein.

Even though the helix content is a bit more in the human pVHL protein the consequence of it, in overall amino acid sequences alignment and structure formation are demonstrated following in Fig. 9 and Supplementary Fig. 10.

**Fig. 10. f10-gi-21073:**
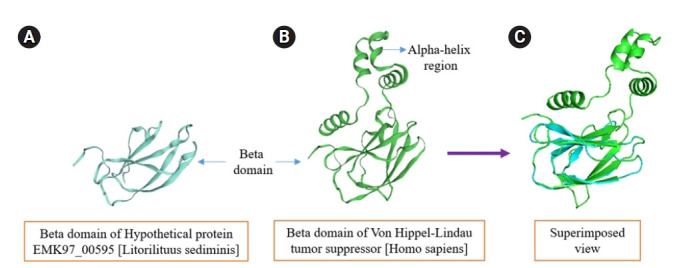
The structural similarity prediction between query and target pVHL protein. (A, B) pVHL proteins contain the beta domain that actually paly role as a tumor suppressor protein is superimposed (using PyMOL) to infer how much structural similarity they have, the superimposed result (C) is absolutely congruous each other in the β domain region which dictate the human pVHL proteins can play magnificent role as a tumor suppressor protein even though it contain α domain. pVHL, von Hippel-Lindau tumor suppressor protein.

The human pVHL protein has a greater instability index than the novel bacterial protein, indicating that the bacterial pVHL protein will be very effective as an anti-proliferative drug to substitute in humans, which necessitates additional research (Fig. 10).

## Discussion

The sequence information as well as the structural information contributes to understanding the function of a hypothetical protein (Tables 1 and 2, Fig. 2, Supplementary Table 1). This study aims to characterize a hypothetical protein, which showed strong homology with VHL superfamily, involved in tumor suppressor. Therefore, the amino acid sequence of the hypothetical protein EMK97_00595 (*Litorilituus sediminis*) was retrieved (Supplementary Table 2), and initially, the physicochemical properties were obtained by ExPASy’s ProtParam tool and the prediction results are the deciding factors for the hydrophilicity, stability, and function of the protein [61]. The protein was considered as a stable one even in a wide temperature range as the instability index and the aliphatic index were 32.71 and 77.37, respectively. And the query protein seems to be hydrophilic as the GRAVY was ‒0.261 (Table 3).

Protein structure is closely associated with its function. The secondary structure, viz. helix, sheet, turn and therefore the coil of any protein has an excellent association with the structure, function, and interaction of the protein (Fig. 3). The query hypothetical protein contains the percentages of alpha-helix, beta-turn, extended strand, and the random coil 21.13%, 9.91%, 33.33%, and 36.15%, respectively (Supplementary Table 3, Supplementary Figs. 1-4). Findings from SOPMA revealed that the protein has an abundance of coiled regions that contributes to higher stability and conservation of the protein structure (Fig. 3) [61]. Moreover, the protein features a reliable helices percentage in its structure, which may facilitate folding by providing more flexibility to the structure; thus, protein interactions could be increased [62].

For the prediction of the protein 3D model, HHpred was employed, where the highest identical template was selected for getting an acceptable model. The query protein WP_012259469.1 showed the highest template identity of 25% with von Hippel-Lindau disease tumor suppressor; E3 ubiquitin ligase, transcription factor, hypoxic signaling, transcription; (*Homo sapiens*) with lowest E-value: 1.1e-11. Ramachandran plot analysis revealed that 91.1% of residues were located in the most favored regions. Moreover, residues in additional allowed regions and generously allowed regions were 7.1% and 0.0%, respectively, which evaluated the quality of the model to be good and reliable as it is generally accepted that if 90% of residues are in the most favored regions, it is likely to be a reliable model [63], shown in Fig. 4B. The model is compatible with its sequence as Verify 3D analysis implies that 93.75% of the residues had an average 3D–1D score of ≥0.2 (Fig. 5).“Overall quality factor” was estimated by ERRAT, which is used to evaluate the amino acid environment for non-bonded atomic interactions. Higher scores indicate higher quality, and the query protein’s quality factor was 60.7143, which is greater than the generally accepted range (>50) for a high-quality model [64]. The average G-factor of the query protein is ‒0.20 obtained from ProFunc analysis, which indicates a usual protein model.

Protein’s active site was determined by CASTp, containing 11 amino acids (F, V, Y, Y, T, L, E, V, T, Q, W) of an area with 52.957 and a volume of 22.609, shown in Fig. 6A and 6B. The subcellular localization obtained from CELLO, BUSCA, and other similar servers, seems to be an extracellular secretory signal peptide (Supplementary Fig. 6) and non-transmembrane (Table 5). As the functions of secreted proteins are diverse, the query hypothetical protein may work like paracrine, autocrine, endocrine, or neuroendocrine depending on the target [65]. Solubility is the most important factor and an excellent index for protein functionality (Supplementary Fig. 5). Protein-sol and SOSUI both predict the hypothetical protein as a soluble one, so it may possess good dispersibility and lead to the formation of finely dispersed colloidal systems.

The superfamily, family, and domain information have been determined by a combinational sequence and structural informative approach based on the e-value of different sequence and structure analysis servers. These servers suggested the following hypothetical protein EMK97_00595 from the organism *L. sediminis* to be a VHL beta domain from the VHL superfamily (Table 6, Supplementary Figs. 8 and 9). VHL tumor suppressor protein can play a role in tumor suppression in multiple ways and the most common of them is targeting the HIF that mediated tumor suppression activity through polyubiquitylation and proteasomal degradation [66]. The major contribution of pVHL is to suppress clear-cell renal cell carcinoma in kidney cancer [66,67] and phosphodiesterase 9A gene as novel biomarker in human colorectal cancer [68].

*L. sediminis* is a novel species and the investigated protein EMK97_00595 is also novel so there is no specific STRING derived protein-protein network is available for this organism. The protein-protein interaction network analysis shown here from *H. sapiens* is just for a supposition model to evaluate how the protein interacted in humans (Fig. 8). The protein-protein interaction of VHL-HIF1A with a combined score of 0.999 indicated a strong relationship between these two proteins. The interaction between VHL and HIF1A indicating the involvement of the same pathway to suppress tumor activity (Table 7, Supplementary Fig. 11) [13].

Overall, the combinational strategy of computing physicochemical properties, evaluating the secondary structure and tertiary structure information, and domain information analysis denoted the protein as VHL tumor suppressor protein that is associated with VHL disease (Table 8, Supplementary Figs. 10, 11).

Protein is the building block of life that serves both biological processes and molecular functions in living organisms. Hence, this study investigated the functional role of a hypothetical protein from a novel bacterium, *L. sediminis* that possesses a significant tumor suppression activity. The employment of highly recommended bioinformatics tools to analyze the combinational sequence and structural information revealed the underlying molecular function of the examined hypothetical protein. The current investigation suggested that the hypothetical protein may exhibit a VHL beta domain that is similar to the human VHL beta domain and is also a part of pVHL (Figs. 9 and 10). Therefore, this finding with the aid of bioinformatics tools can soften our viewpoint for further investigation and experimental validation of this hypothetical protein containing VHL beta domain, and the use of this hypothetical protein with the aid of modern biotechnology might be utilized to suppress tumor progression in higher organisms such as human as an alternative to human defective or mutated VHL protein in the near future.[Fig f1-gi-21073][Fig f10-gi-21073]

## Figures and Tables

**Table 1.** Similar proteins obtained from the non-redundant database

Accession No.	Description	Scientific name	Total score	Query cover (%)	E-value	Identity (%)
WP_118961164.1	Hypothetical protein (*Colwellia* sp. RSH04)	*Colwellia* sp. RSH04	349	100	5.00E-120	74.18
WP_033081725.1	Hypothetical protein (*Colwellia psychrerythraea*)	*Colwellia psychrerythraea*	235	100	4.00E-75	51.17
WP_142932219.1	Hypothetical protein (*Aliikangiella* sp. M105)	*Aliikangiella* sp. M105	108	94	2.00E-25	34.78
WP_155746905.1	Hypothetical protein (*Scytonema* sp UIC 10036)	*Scytonema* sp. UIC 10036	61.2	45	3.00E-08	34.02
BAZ36602.1	Hypothetical protein NIES4101_25210 (*Calothrix* sp NIES-4101)	*Calothrix* sp. NIES-4101	57.8	27	5.00E-07	44.83

**Table 2.** Similar proteins obtained from Swissprot database

Entry	Protein names	Identity (%)	Score	E-value
A0A396TZK2	Uncharacterized protein (*Colwellia* sp. *RSH04*)	74.2	894	1.3e-120
A0A545UCJ6	VHL domain-containing protein (*Aliikangiella* sp. *M105*)	34.3	81	8.3e-28
A0A1Z4R2C0	VHL domain-containing protein (*Calothrix *sp. *NIES-4101*)	36.6	150	1.5e-9
A0A1I6H391	Por secretion system C-terminal sorting domain-containing protein (*Robiginitalea myxolifaciens*)	37.1	133	7e-6
A0A2S7JPT4	VHL domain-containing protein (*Limnohabitans *sp. *TS-CS-82*)	35.1	124	2e-5

**Table 3.** Physicochemical properties of the hypothetical protein (WP_130598461.1)

Property	Value
Molecular weight	23,229.44
Theoretical pI	4.22
Total No. of negatively charged residues (Asp + Glu)	27
Total No. of positively charged residues (Arg + Lys)	10
The instability index (II) is computed to be	32.71
Formula	C_1024_H_1552_N_262_O_346_S_5_
Total No. of atoms	3189
Aliphatic index	77.37
Grand average of hydropathicity (GRAVY)	‒0.261

**Table 4.** Ramachandran plot statistics of the predicted 3D model for the target protein EMK97_00595 (WP_130598461.1)

Plot statistics	No. of amino acid residues (%)
Residues in the most favored regions [A, B, L]	51 (91.1)
Residues in additional allowed regions [a, b, l, p]	4 (7.1)
Residues in generously allowed regions [~a, ~b, ~l, ~p]	0
Residues in disallowed regions	1 (1.8)
No. of non-glycine and non-proline residues	56 (100)
No. of end-residues (excl. Gly and Pro)	2
No. of glycine residues (shown as triangles)	4
No. of proline residues	2
Total No. of residues	64

**Table 5.** Assessment of subcellular localization

Prediction	Servers	Results
Prediction of subcellular localization	BUSCA	Extracellular space, signal peptide
	Cello	Extracellular
	PsortB	Unknown, signal peptide
	Cell-PLoc	Extracellular
	PSLpred	Extracellular protein
	SOSUIgramN	Outer membrane
Signal peptide prediction	Predisi	Secreted protein, signal peptide
	SignalP-5.0 Server	Signal Peptide
Prediction of protein solubility	SOSUI	Soluble protein
	Protein-sol	Soluble protein
Prediction of transmembrane helices	HMMTOP	None
	TMHMM	None
	Sable	No transmembrane domain

**Table 6.** Function annotation of hypothetical protein through the analysis of protein domain/superfamily/family

Server	Domain/Superfamily/Family	e-value/Confidence	Region/Alignment
Functional annotation from sequence			
Conserved domain database (CDD)	Superfamily: pVHL	6.22e-05	146‒197
Pfam	Family: VHL (VHL beta domain)	1.3e-02	144‒200
SMART	VHL	1.2e-02	133‒205
Interpro	VHL superfamily	-	144‒199
	VHL beta domain	-	131‒212
Superfamily 1.75 (SCOP)	Superfamily: VHL	2.71e-09	144‒199
	Family: VHL	8.1e-03	
Supfam	Superfamily: VHL	1.54e-09	144‒199
	Family: VHL	8.1e-03	
Motif (From Pfam)	VHL beta domain	8.1e-03	146‒200
Functional annotation from the 3D structure			
ProFunc	VHL beta domain	9.11e-05	131‒191
Phyre 2	Superfamily: VHL	99.8% (confidence)	135‒212
	Family: VHL		
CATH-Gene3D (from Interpro)	VHL beta domain	-	131‒212

**Table 7.** Interacting proteins and their combined score from STRING 11.0 server

Interacted protein	Combined score
AKT1 (RAC-alpha serine/threonine-protein kinase)	0.997
AKT2 (RAC-beta serine/threonine-protein kinase)	0.994
CUL2 (cullin-2; core component of multiple cullin-RING-based ECS E3 ubiquitin-protein ligase complexes)	0.999
EGLN1 (Egl nine homolog 1)	0.989
EPAS1 (endothelial PAS domain-containing protein 1)	0.994
HIF1A (hypoxia-inducible factor 1-alpha)	0.999
PPP2CA (serine/threonine-protein phosphatase 2A catalytic subunit alpha isoform)	0.993
RBX1 (E3 ubiquitin-protein ligase RBX1)	0.982
TCEB1 (elongin-C)	0.999
TCEB2 (elongin-B)	0.998

**Table 8.** Comparison between query and target pVHL protein properties

Characteristics of pVHL protein	Litorilituus sediminis	*Homo sapiens*
No. of residues	213	213
Molecular weight	23,229.44	24,152.78
Theoretical pI	4.22	4.68
Aliphatic index	77.37	75.45
Overall confidence value (%)	75.4	78.2
Predicted % helix content	11 (24 residues)	28 (60 residues)
Predicted % beta sheet content	43 (91 residues)	12 (26 residues)
Predicted % voil content	46 (98 residues)	60 (127 residues)
Instability index	32.71	68.65

pVHL, von Hippel-Lindau tumor suppressor protein.

## Supplementary Materials

Supplementary Table 1. Information of query hypothetical protein

Supplementary Table 2. Total number of amino acid composition and percentages

Supplementary Table 3. Components of secondary structure of query hypothetical protein

Supplementary Fig. 1. Amino acid sequence from SOPMA.

Supplementary Fig. 2. Secondary structure plot of VHL domain. VHL, von Hippel-Lindau.

Supplementary Fig. 3. PSIPRED shows the helix (red) and strand (green) positions in sequence.

Supplementary Fig. 4. PCoils represents the position of Window 14 (green), Window 21 (blue), and Window 28 (purple) in the sequence.

Supplementary Fig. 5. ERRAT generated overall quality factor bar diagram.

Supplementary Fig. 6. Conserved domain of VHL superfamily. VHL, von Hippel-Lindau.

Supplementary Fig. 7. Result of Motif finder.

Supplementary Fig. 8. Polarity prediction result of PSIPRED.

Supplementary Fig. 9. Signal peptide prediction of WP_130598461.1 through SignalP-5.0.

Supplementary Fig. 10. Amino acid and secondary structure comparison between hypothetical and human pVHL protein. pVHL, von Hippel-Lindau tumor suppressor protein.

Supplementary Fig. 11. Mechanism of pVHL mediated tumor suppression activity. The pVHL comprises a small α and a large β subunit. The α-domain serves as a binding site, whereas the β-domain plays important role in substrate recognition. During normoxia (oxygen available condition), HIFα binds with VHL beta domain and VHL alpha domain associates with E3 ubiquitin ligase via elongin BC complex which leads to efficient ubiquitylation and proteasomal degradation of HIFα that suppress tumor proliferation activity. On the other hand, During Hypoxia or when pVHL is defective, HIFα unable to recognize pVHL and so promotes tumor progression process. pVHL, von Hippel-Lindau tumor suppressor protein; HIFα, hypoxia inducing factor-α.
