# Supplementary material for: Hypothetical protein predicted to be tumor suppressor: a protein functional analysis
Source: Genomics Inform. 2022 Mar 31;20(1):e6. doi: 10.5808/gi.21073 (PMC9002001; doi:10.5808/gi.21073)
Supplement: Supplementary Table 1. — Information of query hypothetical protein [file gi-21073-suppl1.pdf]

Supplementary Table 1. Information of query hypothetical protein

Query protein	Description
Definition	Hypothetical protein
Accession	WP_130598461
Version	WP_130598461.1
No. of amino acid residues	213
Locus	WP_130598461
Source databases	RefSeq and GenBank
Source organism	*Litorilituus sediminis*<br>(Gram-negative bacteria)
