# Supplementary material for: Hypothetical protein predicted to be tumor suppressor: a protein functional analysis
Source: Genomics Inform. 2022 Mar 31;20(1):e6. doi: 10.5808/gi.21073 (PMC9002001; doi:10.5808/gi.21073)
Supplement: Supplementary Table 2. — Total number of amino acid composition and percentages [file gi-21073-suppl2.pdf]

Supplementary Table 2. Total number of amino acid composition and percentages

Amino acid composition	Total count	Percentages
Ala (A)	17	8.00
Arg (R)	5	2.30
Asn (N)	13	6.10
Asp (D)	14	6.60
Cys (C)	4	1.90
Gln (Q)	7	3.30
Glu (E)	13	6.10
Gly (G)	15	7.00
His (H)	3	1.40
Ile (I)	16	7.50
Leu (L)	10	4.70
Lys (K)	5	2.30
Met (M)	1	0.50
Phe (F)	5	2.30
Pro (P)	8	3.80
Ser (S)	21	9.90
Thr (T)	22	10.30
Trp (W)	3	1.40
Tyr (Y)	15	7.00
Val (V)	16	7.50
Pyl (O)	0	0.00
Sec (U)	0	0.00
