# Supplementary material for: Hypothetical protein predicted to be tumor suppressor: a protein functional analysis
Source: Genomics Inform. 2022 Mar 31;20(1):e6. doi: 10.5808/gi.21073 (PMC9002001; doi:10.5808/gi.21073)
Supplement: Supplementary Table 3. — Components of secondary structure of query hypothetical protein [file gi-21073-suppl3.pdf]

Supplementary Table 3. Components of secondary structure of query hypothetical protein

Components of secondary structure	Colored symbol	No. of components	Percentages of components
Alpha helix	(Hh)	45	21.13
3_{10} helix	(Gg)	0	0.00
Pi helix	(Ii)	0	0.00
Beta bridge	(Bb)	0	0.00
Extended strand:	(Ee)	71	33.33
Beta turn	(Tt)	20	9.91
Bend region	(Ss)	0	0.00
Random coil	(Cc)	77	36.15
Ambiguous states	(?)	0	0.00
Other states	-	0	0.00
