# Supplementary material for: Hypothetical protein predicted to be tumor suppressor: a protein functional analysis
Source: Genomics Inform. 2022 Mar 31;20(1):e6. doi: 10.5808/gi.21073 (PMC9002001; doi:10.5808/gi.21073)
Supplement: Supplementary Fig. 1. — Amino acid sequence from SOPMA. [file gi-21073-suppl4.pdf]

```
        10        20        30        40        50        60        70
         |         |         |         |         |         |         |
MSNKLASVLVFALTSFASQQSFAEQCYVETSTGGGPSGPATCIDISVDNIDGTSVSLSATAVRYGDDISN
hhhhhhhheeehhhhhhhhhhhccceeeecccccccccccceeeeeeectttcceeeehhhhettcccch
YIIWSSDIDGNIGYGTSTVAELTPGTHKVTAVAQVPHLRPYIDEETIVIASPEDTSCSNIAPSTAETFDE
eeeeeccccccccccchhhhhccttcceeeeeecccccccccttheeeeeccccccccccchhhhhhhhh
YYGIEFTNNRESDIKVYWLKYENAERIYYYTLAQGERVTQTGYPGNKWLVTDANDNCLSVHTNGYQADYV
hheeeecccccccեeeeeeeecctthhheeeeehcttcceeetccttceeeeeettcceeeeeettccctee
TIN
eee
```

Supplementary Fig. 1. Amino acid sequence from SOPMA.
