# Supplementary figures and images for: Hypothetical protein predicted to be tumor suppressor: a protein functional analysis
Source: Genomics Inform. 2022 Mar 31;20(1):e6. doi: 10.5808/gi.21073 (PMC9002001; doi:10.5808/gi.21073)

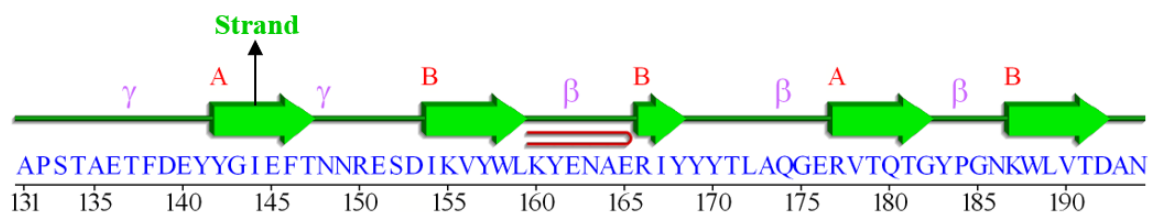

**Supplementary Fig. 2.** Secondary structure plot of VHL domain. VHL, von Hippel-Lindau.

Supplement: Supplementary Fig. 2. — Secondary structure plot of VHL domain. VHL, von Hippel-Lindau. [file gi-21073-suppl5.pdf]

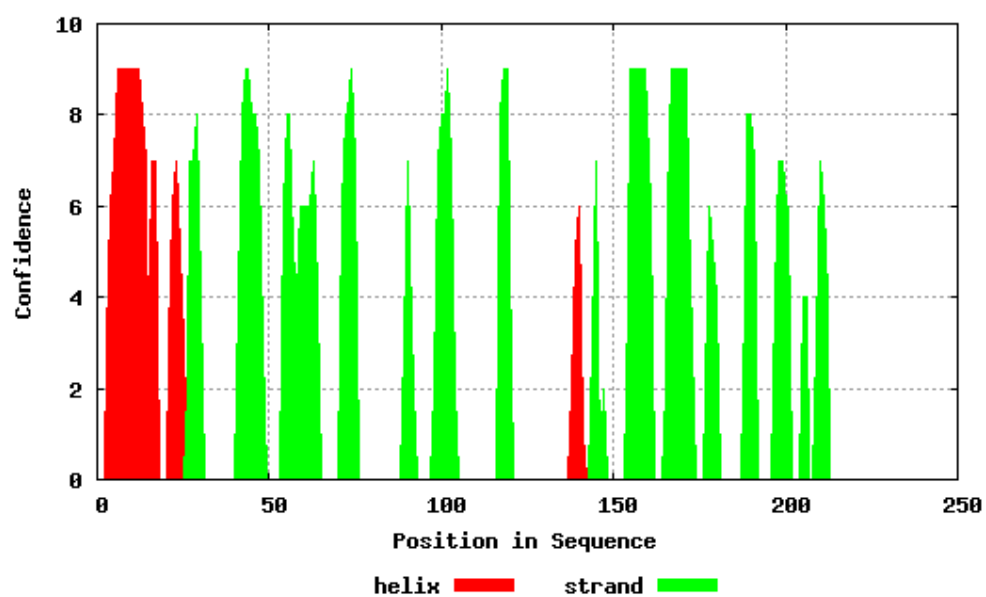

**Supplementary Fig. 3.** PSIPRED shows the helix (red) and strand (green) positions in sequence.

Supplement: Supplementary Fig. 3. — PSIPRED shows the helix (red) and strand (green) positions in sequence. [file gi-21073-suppl6.pdf]

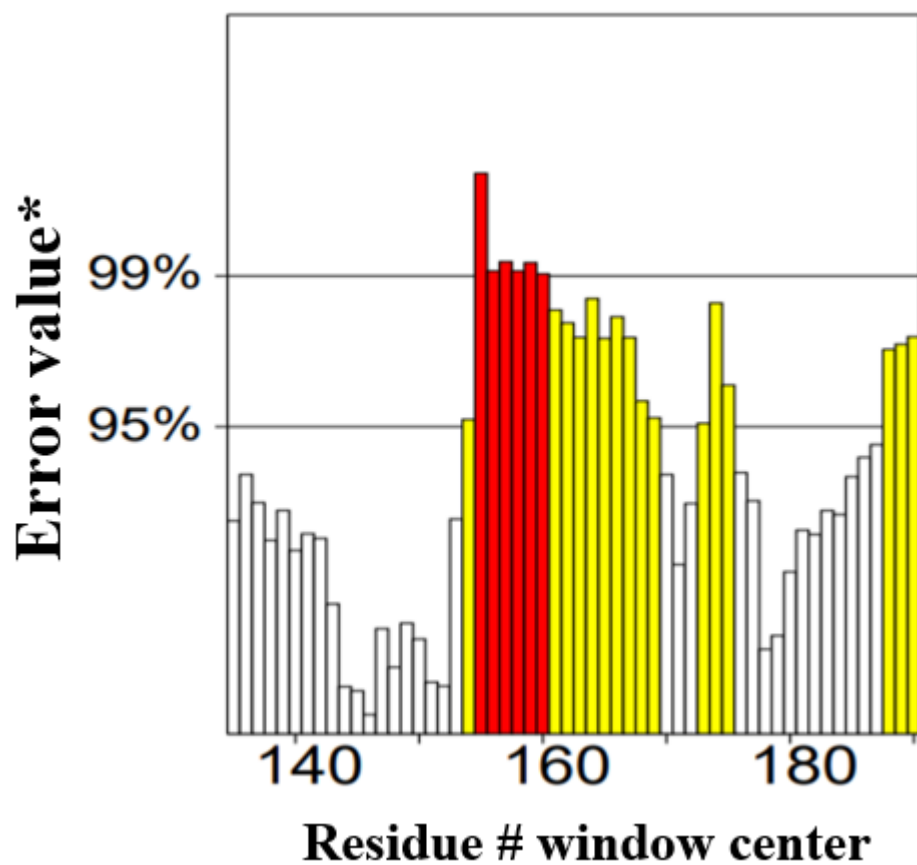

**Supplementary Fig. 5.** ERRAT generated overall quality factor bar diagram.

Supplement: Supplementary Fig. 5. — ERRAT generated overall quality factor bar diagram. [file gi-21073-suppl8.pdf]

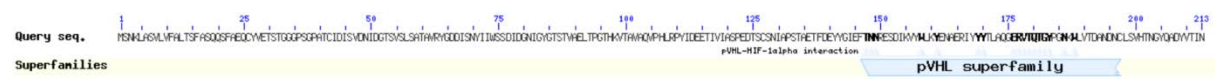

**Supplementary Fig. 6.** Conserved domain of VHL superfamily. VHL, von Hippel-Lindau.

Supplement: Supplementary Fig. 6. — Conserved domain of VHL superfamily. VHL, von Hippel-Lindau. [file gi-21073-suppl9.pdf]

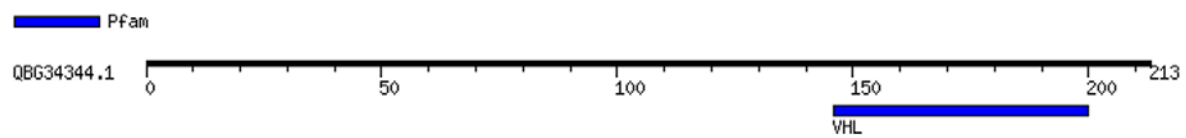

**Supplementary Fig. 7.** Result of Motif finder.

Supplement: Supplementary Fig. 7. — Result of Motif finder. [file gi-21073-suppl10.pdf]

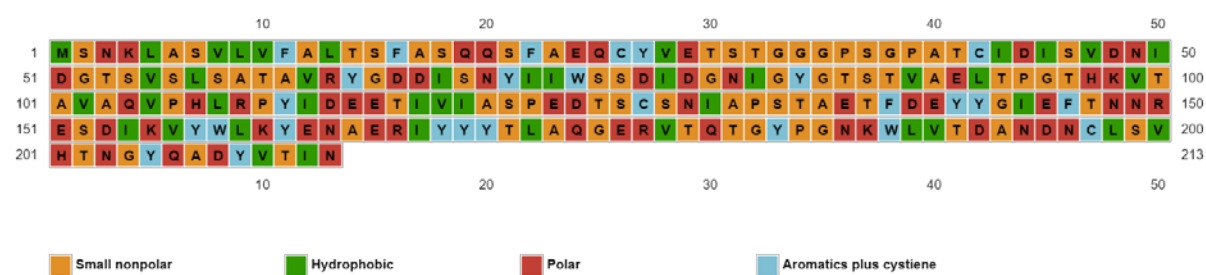

**Supplementary Fig. 8.** Polarity prediction result of PSIPRED.

Supplement: Supplementary Fig. 8. — Polarity prediction result of PSIPRED. [file gi-21073-suppl11.pdf]

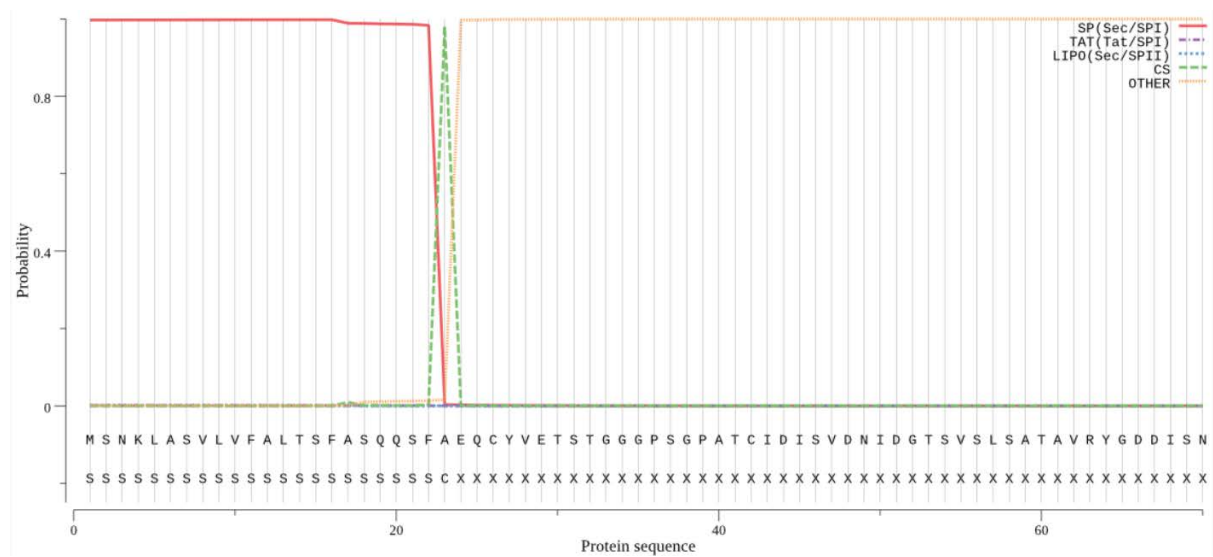

**Supplementary Fig. 9.** Signal peptide prediction of WP\_130598461.1 through SignalP-5.0.

Supplement: Supplementary Fig. 9. — Signal peptide prediction of WP_130598461.1 through SignalP-5.0. [file gi-21073-suppl12.pdf]
