# Supplementary material for: Hypothetical protein predicted to be tumor suppressor: a protein functional analysis
Source: Genomics Inform. 2022 Mar 31;20(1):e6. doi: 10.5808/gi.21073 (PMC9002001; doi:10.5808/gi.21073)
Supplement: Supplementary Fig. 4. — PCoils represents the position of Window 14 (green), Window 21 (blue), and Window 28 (purple) in the sequence. [file gi-21073-suppl7.pdf]

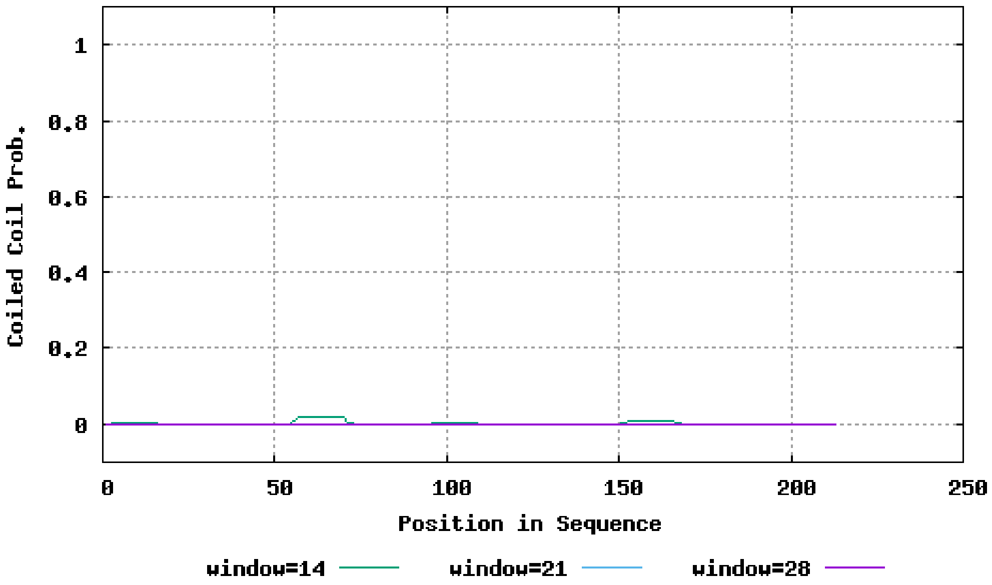

Supplementary Fig. 4. PCoils represents the position of Window 14 (green), Window 21 (blue), and Window 28 (purple) in the sequence.
