# Supplementary material for: Hypothetical protein predicted to be tumor suppressor: a protein functional analysis
Source: Genomics Inform. 2022 Mar 31;20(1):e6. doi: 10.5808/gi.21073 (PMC9002001; doi:10.5808/gi.21073)
Supplement: Supplementary Fig. 10. — Amino acid and secondary structure comparison between hypothetical and human pVHL protein. pVHL, von Hippel-Lindau tumor suppressor protein. [file gi-21073-suppl13.pdf]

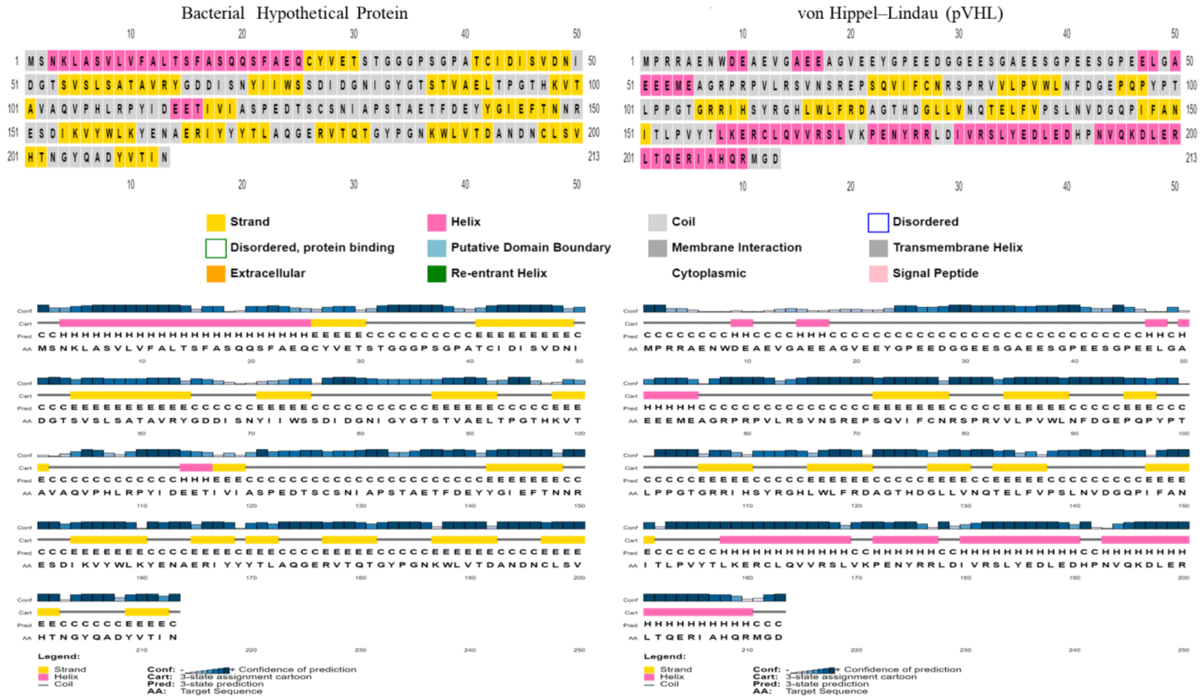

Supplementary Fig. 10. Amino acid and secondary structure comparison between hypothetical and human pVHL protein. pVHL, von Hippel-Lindau tumor suppressor protein.
