# Supplementary material for: Hypothetical protein predicted to be tumor suppressor: a protein functional analysis
Source: Genomics Inform. 2022 Mar 31;20(1):e6. doi: 10.5808/gi.21073 (PMC9002001; doi:10.5808/gi.21073)
Supplement: Supplementary Fig. 11. — Mechanism of pVHL mediated tumor suppression activity. The pVHL comprises a small α and a large β subunit. The α-domain serves as a binding site, whereas the β-domain plays important role in substrate recognition. During normoxia (oxygen available condition), HIFα binds with VHL beta domain and VHL alpha domain associates with E3 ubiquitin ligase via elongin BC complex which leads to efficient ubiquitylation and proteasomal degradation of HIFα that suppress tumor proliferation activity. On the other hand, During Hypoxia or when pVHL is defective, HIFα unable to recognize pVHL and so promotes tumor progression process. pVHL, von Hippel-Lindau tumor suppressor protein; HIFα, hypoxia inducing factor-α. [file gi-21073-suppl14.pdf]

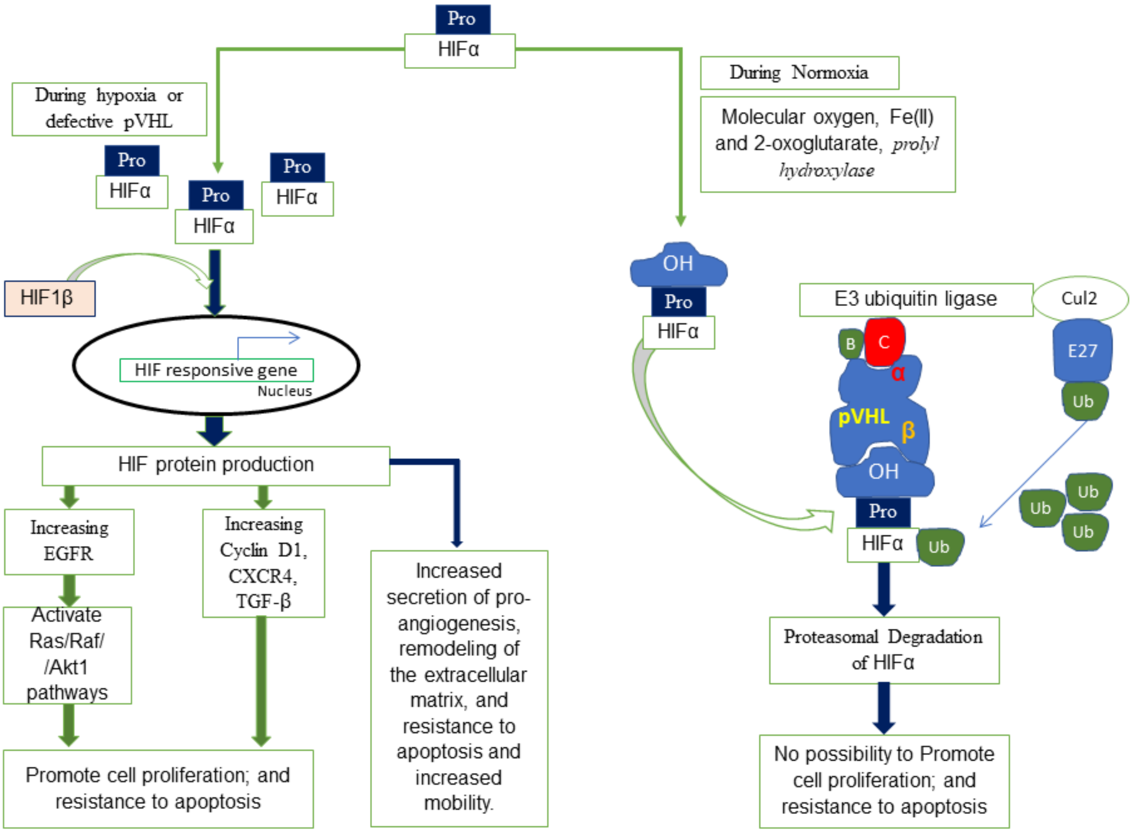

Supplementary Fig. 11. Mechanism of pVHL mediated tumor suppression activity. The pVHL comprises a small α and a large β subunit. The α-domain serves as a binding site, whereas the β-domain plays important role in substrate recognition. During normoxia (oxygen available condition), HIFα binds with VHL beta domain and VHL alpha domain associates with E3 ubiquitin ligase via elongin BC complex which leads to efficient ubiquitylation and proteasomal degradation of HIFα that suppress tumor proliferation activity. On the other hand, During Hypoxia or when pVHL is defective, HIFα unable to recognize pVHL and so promotes tumor progression process. pVHL, von Hippel-Lindau tumor suppressor protein; HIFα, hypoxia inducing factor-α.
